# Heterogeneity in past-year smoking, current tobacco use, and smoking cessation behaviors among light and/or non-daily smokers

**DOI:** 10.18332/tid/125724

**Published:** 2020-09-04

**Authors:** Toluwa Omole, Timothy McNeel, Kelvin Choi

**Affiliations:** 1 Division of Intramural Research, National Institute on Minority Health and Health Disparities, Bethesda, United States; 2 Information Management Services Inc., Rockville, United States

**Keywords:** smoking cessation, light daily smoking, non-daily smoking

## Abstract

**INTRODUCTION:**

Prevalence of light daily smoking, <10 cigarettes per day (CPD), and non-daily smoking has increased in the US population. This analysis examined the heterogeneity in past-year smoking behavior, current tobacco use behaviors, and smoking cessation behaviors among light and/or non-daily smokers.

**METHODS:**

Current adult (≥18 years old) smokers (N=26196) participated in the 2010–2011 US Current Population Survey – Tobacco Use Supplement, which reported current (T1) and past 12-month (T0) smoking behaviors. Responses were categorized by intensity (light ≤10 CPD vs heavy >10 CPD) and frequency (non-daily vs daily). Combinations of T0 and T1 smoking behaviors resulted in 15 smoking trajectories ending in light/non-daily smoking and a 16th category of heavy daily smokers at T1. Differences in demographics, tobacco use, and smoking cessation behaviors were assessed by using weighted multivariable regression models.

**RESULTS:**

Overall, 46.1% of US smokers were heavy smokers, 24.6% remained light daily smokers and 12.5% remained light non-daily smokers between T0 and T1. Current cigar, smokeless tobacco, and pipe use differed by smoking trajectories (p<0.05). All light and/or non-daily smokers were more likely than heavy daily smokers to have made a quit attempt (p<0.05) but use of cessation treatments varied. Smokers in many light and/or non-daily smoking trajectories were less likely than heavy daily smokers to be aided by healthcare providers for smoking cessation (p<0.05).

**CONCLUSIONS:**

Among heavy daily smokers who became light non-daily smokers, the mismatch between intent to quit (80.9%) and receiving advice to set a quit date (33.7%) is one example of a potential opportunity for a clinical intervention.

## INTRODUCTION

From 2005 through 2016 the prevalence of heavy daily smoking, >10 cigarettes per day (CPD), and more specifically ≥20 CPD among the US population decreased, while the prevalence of light daily smoking (≤10 CPD) among the US population increased^1^. Additionally, the decline in the prevalence of non-daily smoking (also known as intermittent smoking) was slower than the decline in prevalence of daily smoking in the US population, resulting in an increase in the proportion of non-daily smoking among current smokers, and has varied by racial/ethnic group over the last decade^2^. Specifically, among non-Hispanic White smokers the prevalence of non-daily smoking has decreased from 11% to 10%, among non-Hispanic Black smokers the prevalence has decreased from 13% to 11%, and among Hispanic smokers the prevalence has decreased from 13% to 8%^2^. Despite the decline in both daily and non-daily smoking, racial/ethnic minorities are more likely to be light or non-daily smokers compared to White smokers^3,4^. Previous research suggests that intermittent smokers score lower on the Fagerström test for nicotine dependence (FTND), which indicates that these smokers are less dependent on nicotine than daily smokers^5-7^.

Much less is known about the intersection between frequency of smoking (i.e. daily/non-daily) and intensity of smoking (i.e. heavy/light). For example, some intermittent smokers used to be daily smokers while others never were daily smokers^7,8^. Additionally, despite the differences between daily smokers and intermittent smokers, approximately 70% to 80% of the quit attempts of intermittent smokers are still unsuccessful, while over 85% of daily smoker attempts are unsuccessful^9^. Prior research suggests that nicotine dependence is lower among light non-daily smokers compared to light daily smokers^10^. Nonetheless, among smokers who smoke their first cigarette within 5 minutes after waking (indication of nicotine dependence), 25% are light non-daily smokers^10^. An additional limitation in the literature is that most of the previous reports on frequency and intensity of cigarette smoking are based on current use, while light daily smoking and non-daily smoking could be a transitional stage for those who just started smoking, or for those who are trying to quit smoking by cutting back cigarette consumption^7,8^. No studies, to date, have examined how light/heavy and daily/non-daily smoking are associated with receiving advice from healthcare providers to quit smoking and cessation treatment use. Additionally, light and intermittent smoking still carry significant health risks, therefore understanding this increasingly prevalent behavior among smokers can inform strategies to reduce tobacco induced diseases.

Consequently, the current analysis aim is to provide a detailed examination of light daily smoking and non-daily smoking. Specifically, the first aim of this analysis is to estimate the prevalence of different smoking groups that resulted in light daily smoking and non-daily smoking. The second aim is to examine the changes in cigarette consumption over a year within these groups as well as compare them with those in heavy daily smokers. The third aim is to examine the use of other tobacco products by these groups. Lastly, the fourth aim is to examine the associated smoking cessation history and receipt of smoking cessation advice by these groups. Given the health risks (e.g. for cardiovascular disease and cancer^11,12^ and overall mortality^13^) associated with light daily smoking and non-daily smoking, better understanding of tobacco use and cessation behaviors among light daily smokers and non-daily smokers compared to heavy daily smokers may help to identify potential interventions that could lead to better health outcomes.

## METHODS

### Study population

Data were obtained from the Tobacco Use Supplement to the Current Population Survey (TUS-CPS). TUS-CPS is sponsored by the National Cancer Institute and is conducted along with the Current Population Survey (CPS) by the Census Bureau, which provides information on demographic characteristics and employment. The sampling frame of the TUS-CPS is the non-institutionalized civilian population of the United States. Regarding the sampling process of the CPS, the United States is divided into 2025 primary sampling units (PSU), which are then grouped into 824 strata. Afterwards, within each of the strata one PSU is chosen and households within the chosen PSUs are surveyed. This analysis was based on data from the 2010–2011 TUS-CPS, which was composed of about 83000 respondents, representing the non-institutionalized population of civilians aged ≥18 years. We did not use more recent data (e.g. 2014–2015 TUS-CPS) because they did not assess interactions with healthcare providers about smoking cessation and use of cessation treatment. The analysis was limited to self-respondents because proxy respondents did not provide detailed tobacco use information. Of those, 68% of the self-responses were from telephone interviews, while 32% of the self-responses were from personal interviews, with an overall response rate of 59.8%. Because the research questions focused on smoking behaviors, the overall analytic sample was further restricted to current smokers (reported smoking every day or some days) at the time of survey who had smoked at least 100 cigarettes in their lifetime (n=26196).

### Measures

Respondents were asked: ‘Do you now smoke cigarettes every day, some days, or not at all?’. Their response to this question categorized them initially as every-day or some-day smokers. For the purposes of this analysis, current every-day smokers were classified as daily smokers and current some-day smokers were classified as non-daily smokers. Respondents were also asked: ‘On average, how many cigarettes do you now smoke each day?’ and ‘Around this time 12 months ago, on average, about how many cigarettes did you smoke each day?’. Based on prior literature^10,14^, if current smokers reported smoking ≤10 CPD, they were classified as light smokers, whereas if they smoked >10 CPD they were classified as heavy smokers. The combination of daily/non-daily and light/heavy smoking status yielded four main smoking categories at the time of survey (T1): light daily (LD), heavy daily (HD), light non-daily (LND) and heavy non-daily (HND) smoking. Respondents also reported their smoking status (every day, some days, not at all) and CPD 12 months prior to the survey (T0). Smoking status and CPD at T0 were used to create 5 smoking categories of non-smoking (NS), LD, HD, LND, and HND. Finally, smoking categories at T0 and T1 were used to create 15 groups from T0 to T1 that resulted in light and/or intermittent smoking at T1, and a 16th group of respondents that represented current heavy daily smokers at T1, which served as the reference group. Most smokers (90%) in the reference group were heavy daily smokers at T0 as well (Table 1).

**Table 1:** Weighted distributions of demographic characteristics by smoking groups (N=26196)

	*Overall*	*Light daily to light daily (n=6378)*	*Heavy daily to light daily (n=1357)*	*Light non-daily to light daily (n=604)*	*Heavy non-daily to light daily (n=52)*	*Non-smoker to light daily (n=392)*	*Light daily to light non-daily (n=448)*	*Heavy daily to light non-daily (n=293)*	*Light non-daily to light non-daily (n=3081)*	*Heavy non-daily to light non-daily (n=112)*	*Non-smoker to light non-daily (n=760)*	*Light daily to heavy non-daily (n=17)*	*Heavy daily to heavy non-daily (n=97)*	*Light non-daily to heavy non-daily (n=45)*	*Heavy non-daily to heavy non-daily (n=127)*	*Non-smoker to heavy non-daily (n=35)*	*Current heavy daily (n=12398)*
	*%*	*%*	*%*	*%*	*%*	*%*	*%*	*%*	*%*	*%*	*%*	*%*	*%*	*%*	*%*	*%*	*%*
**Sex**																	
Male	54.1	44.6	48.3	47.8	62.0	43.6	45.0	53.3	59.2	59.5	51.6	67.2	66.3	65.1	74.3	54.2	59.1
Female	45.9	55.4	51.7	52.2	38.0	56.4	55.0	46.7	40.8	40.5	48.4	32.8	33.7	34.9	25.7	45.8	40.9
**Age** (years)																	
18–34	35.2	39.9	43.2	51.0	47.0	50.3	50.9	35.8	43.6	33.5	51.0	26.3	36.0	30.3	27.3	26.2	26.7
35–49	29.5	28.7	23.7	25.1	18.4	17.8	21.2	31.2	29.9	32.5	23.9	17.9	22.1	19.9	25.9	20.7	32.0
≥50	35.2	31.4	33.1	23.9	34.6	31.9	27.9	33.0	26.5	34.0	25.1	55.7	41.9	49.7	46.8	53.1	41.3
**Race/ethnicity**																	
Hispanic	9.4	12.5	6.1	14.8	35.2	12.9	17.4	3.5	19.9	13.0	18.0	23.3	5.1	23.0	9.3	15.7	3.8
NH-White	74.5	64.9	77.3	58.0	47.9	71.4	60.7	79.6	58.2	63.9	64.3	55.3	84.0	57.4	76.8	78.7	86.0
NH-Black	11.0	16.4	9.6	18.3	15.4	11.1	15.9	12.3	15.4	15.9	10.3	21.4	5.2	14.9	11.9	5.6	6.6
NH-AI/AN/HI	1.2	1.3	1.2	1.8	0.2	0.7	0.6	1.7	1.6	0.6	2.4	0	1.8	0.5	1.6	0	0.9
NH-Asian	2.3	3.3	2.6	6.1	1.3	1.8	4.2	1.1	3.3	0.4	2.4	0	0	4.3	0	0	1.2
NH-Multi	1.7	1.7	3.1	1.0	0	2.1	1.1	1.7	1.5	6.2	2.6	0	3.9	0	0.4	0	1.6
**Education**																	
≤ HS education	57.0	55.9	60.6	56.5	59.5	55.5	47.5	53.2	47.3	58.0	40.1	74.1	64.6	71.4	65.7	41.7	61.3
Some college	22.1	22.8	22.0	22.5	26.3	23.2	27.9	23.3	22.6	20.9	26.8	5.7	22.7	18.6	12.8	37.0	21.2
Associate degree	8.8	9.3	7.5	8.7	1.7	8.0	9.2	12.6	10.3	9.0	10.2	0	8.0	5.5	7.8	4.4	8.2
College degree	12.0	12.0	9.9	12.4	12.4	13.3	15.4	10.9	19.8	12.1	22.9	20.2	4.7	4.5	13.7	16.9	9.3
**Family income (US$)**																	
<25000	37.0	38.1	44.0	47.1	36.5	35.7	40.2	39.4	34.3	36.2	33.1	20.6	31.0	53.9	45.3	49.2	35.8
25000–49000	30.6	30.7	29.0	24.6	40.1	35.1	32.1	28.7	28.8	29.4	26.8	47.7	39.0	15.3	22.5	22.8	31.8
50000–74999	16.2	16.0	13.6	13.9	10.5	13.2	14.1	18.8	16.4	18.8	18.5	21.3	12.1	21.5	11.6	12.3	16.7
≥75000	16.2	15.2	13.4	14.3	12.8	16.0	13.6	13.1	20.6	15.6	21.7	10.4	17.9	9.3	20.6	15.6	15.7

NH: non-Hispanic. HS: high school.

Respondents were asked: ‘On how many of the past 30 days did you smoke?’, which was used in the calculation for cigarettes smoked per month (CPM). To calculate CPM at T0 and T1, among every-day smokers, CPD was multiplied by 30 days; among non-daily smokers, CPD was multiplied by number of days smoked in the past 30 days. To calculate changes in cigarettes smoked per month, CPD at T1 was subtracted from CPD at T0. Regarding non-cigarette tobacco product use, respondents reported if they used the following products every day, some days, or not at all, at the time of survey: regular cigars, cigarillos, regular pipes, hookah, or smokeless tobacco products. Respondents who reported every-day or some-day use were classified as current users of that product.

In terms of smoking cessation, respondents reported if they attempted to quit smoking for any length of time during the 12 months prior to the time of survey (yes/no). Among those who reported making a quit attempt, respondents reported using of a variety of cessation treatment methods such as nicotine replacement therapy, prescription medication, and counseling. Further, among the respondents who reported seeing a doctor or a dentist during the 12 months prior to the time of survey, they reported if a doctor or dentist advised them to stop smoking, and if a doctor or a dentist recommended that they use nicotine replacement therapy, prescription medication, counseling programs, or set a specific quit date, to aid their smoking cessation attempt. Advice from both sources was combined to represent healthcare provider recommendations.

Demographic information on age, sex, race, income, and education, was collected. Age was coded into three groups: 18–34, 35–49, and >49 years. Sex was coded as a binary variable with male or female options. Race was classified into 6 categories consisting of: Hispanic, non-Hispanic White, non-Hispanic Black, non-Hispanic American Indian/Alaskan Native/Hawaiian, non-Hispanic Asian, and Non-Hispanic-Multi race. Educational attainment was classified into 4 categories consisting of high school degree or less, some college, associate degree, and Bachelor’s degree or higher. Annual household income (US$) was grouped into 4 categories: < 25000; 25000–49999; 50000–74999; and ≥75000.

### Statistical analysis

Weighted multiple logistic regression models were used to assess associations between smoking groups and current cigar use, current pipe use, current hookah use, and current smokeless tobacco use, while controlling for race/ethnicity, income, age, education, and sex. Weighted logistic regression models were used to assess associations between smoking groups and use of cessation treatments among current smokers who made a quit attempt in the past 12 months, as well as the associations between smoking groups and current smokers who were advised by a healthcare provider to quit smoking during the past 12 months using a variety of cessation treatment methods. In all these logistic regression models, heavy daily smokers were used as reference group while controlling for race/ethnicity, income, education, age, and sex. The main analyses were conducted using SAS-callable SUDAAN 11.0.1 to account for the complex sample design, and TUS-CPS self-response replicate weights were used. Pairwise comparison results among smoking groups engaging in light smoking or non-daily smoking were reformatted into a compact letter display using the %MULT macro by Piepho^15^. This project only involved the use of de-identified data, which is considered ‘not human subjects research’ and requires no IRB review or approval per NIH policy and 45 CFR 46.

## RESULTS

More than half (53.9%) of US adult smokers were light daily or non-daily smokers at the time of the survey. The most prevalent of the past 12-month groups among light and/or non-daily smokers was maintaining light daily smoking (24.6%), followed by maintaining light non-daily smoking (12.5%), heavy daily to light daily smoking (5.1%), non-smoking to light non-daily smoking (3.1%), light non-daily to light daily smoking (2.4%), light daily to light non-daily smoking (1.7%), non-smoking to light daily smoking (1.5%), heavy daily to light non-daily smoking (1.0%), maintaining heavy non-daily smoking (0.5%), heavy non-daily to light non-daily smoking (0.4%), heavy daily to heavy non-daily smoking (0.4%), heavy non-daily to light daily smoking (0.2%), light non-daily to heavy non-daily smoking (0.2%), light daily to heavy non-daily smoking (0.1%), and non-smoking to heavy non-daily smoking (0.1%). Less than half (46.1%) were heavy daily smokers.

Table 1 shows the distribution of demographic characteristics for US adult smokers and within each smoking group. The proportion of men was generally higher than that of women in groups resulting in heavy smoking, while the proportion of women was generally higher than that of men in groups resulting in light daily smoking. Younger smokers (aged 18–34 years) were more likely to be in groups with changes in smoking frequency and quantity, while older smokers (aged ≥50 years) were more likely to be current heavy daily smokers. Hispanic and non-Hispanic Black smokers were more likely to be in the groups of light daily to light daily smoking and light non-daily to light non-daily smoking compared to current heavy daily smoking. Smokers with less education (<high school) were more likely to be light daily, non-daily and current heavy daily smokers compared to smokers who had completed some college or had obtained an associate or college degree. Smokers with lower income (<25000 or 25000–49999 US$) were more likely to be light daily, non-daily and current heavy daily smokers, compared to smokers who had higher incomes (50000–74999 and ≥75000 US$). Supplementary Table S1 shows the adjusted odds ratios comparing demographic characteristics of each smoking group to those of heavy daily smokers at T1.

Table 2 displays the tobacco use behaviors by group. Regarding changes in cigarette consumption between T0 and T1, adult smokers reporting decreases in frequency (i.e. daily to non-daily) and/ or quantity (i.e. heavy to light) generally showed reductions in monthly cigarette consumption (e.g. heavy daily to light daily smokers showed an average reduction of 330.1 cigarettes per month). Likewise, those reporting increases in frequency and/or quantity generally showed increases in monthly cigarette consumption (e.g. light non-daily to light daily smokers showed an average increase of 86.8 cigarettes per month). Interestingly, heavy non-daily to light daily smokers showed a reduction in cigarette consumption. Moreover, smokers who maintained light daily smoking and those who maintained light non-daily smoking showed a reduction of 4 cigarettes in monthly cigarette consumption, while those who maintained heavy non-daily smoking showed an increase of 35 cigarettes in monthly cigarette consumption. Compared to current heavy daily smokers, all other groups showed lower odds of smoking their first cigarette <30 minutes after waking; similarly, compared to current heavy daily smokers, all other groups showed lower odds of waking up at night to smoke cigarettes, except for light daily to heavy non-daily smokers. Regarding non-cigarette tobacco product use, compared to current heavy daily smokers, smokers who maintained light daily smoking were less likely to report currently using cigars (6.2% vs 4.1%; adjusted odds ratio, AOR=0.71; 95% CI: 0.58–0.86). In contrast, regarding smokeless tobacco, compared to heavy daily smokers (2.7%), those who maintained light non-daily smoking (4.4%; AOR=1.93; 95% CI: 1.47–2.54), those who changed from non-smoking to light non-daily smoking (4.4%; AOR=2.02; 95% CI: 1.25–3.26), and those who maintained heavy non-daily smoking (9.8%; AOR=3.79; 95% CI: 1.85–7.78) were more likely to report current smokeless tobacco use (data not shown in Table 2).

**Table 2:** Cigarette smoking behaviors and current cigar use by smoking group

*Smoking trajectory*	*Change in cigarettes per month (N=25634)*	*Time to first cigarette ≤30 minutes (N=25996)*		*Awoke at night for cigarette (N=25875)*		*Current cigar use (N=25697)*	
	*Difference*	*Weighted %*	*AOR (95% CI)*	*Weighted %*	*AOR (95% CI)*	*Weighted %*	*AOR (95% CI)*
Light daily to light daily	-3.9^g^	38.1^c^	0.24 (0.22–0.26)	8.0^ce^	0.36 (0.31–0.41)	4.1^b^	0.71 (0.58–0.86)
Heavy daily to light daily	-330.1^k^	48.6^b^	0.36 (0.32–0.41)	15.2^b^	0.78 (0.65–0.93)	5.8^ab^	0.96 (0.70–1.31)
Light non-daily to light daily	86.8^d^	28.1^d^	0.15 (0.12–0.19)	9.2^ce^	0.39 (0.27–0.57)	7.2^a^	1.19 (0.74–1.92)
Heavy non-daily to light daily	-244.8^j^	29.6^cd^	0.18 (0.10–0.34)	3.9^bcf^	0.17 (0.03–0.86)	0.0^c^	-
Non-smoker to light daily	211.1^b^	29.7^d^	0.17 (0.13–0.23)	5.8^cdeg^	0.28 (0.18–0.44)	7.2^a^	1.33 (0.73–2.45)
Light daily to light non-daily	-123.1^h^	16.9^e^	0.08 (0.06–0.11)	8.3^cde^	0.38 (0.23–0.63)	5.7^ab^	0.97 (0.56–1.71)
Heavy daily to light non-daily	-505.5^l^	24.5^de^	0.12 (0.09–0.17)	3.4^df^	0.15 (0.07–0.35)	7.1^ab^	1.12 (0.62–2.04)
Light non-daily to light non-daily	-4.4^g^	6.7f	0.03 (0.03–0.03)	3.5^f^	0.16 (0.12–0.21)	6.3^a^	0.94 (0.76–1.17)
Heavy non-daily to light non-daily	-168.1^i^	20.2^de^	0.10 (0.06–0.16)	4.2^ef^	0.18 (0.07–0.44)	5.5^ab^	0.82 (0.29–2.34)
Non-smoker to light non-daily	41.9^e^	6.1^f^	0.03 (0.02–0.04)	1.7^f^	0.08 (0.04–0.16)	6.9^a^	1.09 (0.76–1.58)
Light daily to heavy non-daily	74.7^cdefg^	27.7^bcde^	0.15 (0.04–0.58)	22.6^abc^	1.34 (0.21–8.43)	15.9^ab^	2.98 (0.22–40.25)
Heavy daily to heavy non-daily	-231.0^j^	42.5^bc^	0.29 (0.19–0.43)	15.3^ab^	0.88 (0.50–1.55)	5.1^ab^	0.69 (0.24–1.97)
Light non-daily to heavy non-daily	198.3^bc^	38.5^bcd^	0.23 (0.10–0.50)	2.4^ef^	0.09 (0.01–0.57)	11.6^ab^	2.02 (0.49–8.35)
Heavy non-daily to heavy non-daily	35.3^ef^	26.3^de^	0.13 (0.08–0.20)	9.1^abde^	0.43 (0.19–1.02)	7.1^ab^	1.00 (0.42–2.39)
Non-smoker to heavy non-daily	338.4^a^	37.3^bcd^	0.23 (0.10–0.57)	0.5^fg^	0.02 (0.00–0.33)	2.7^ab^	0.45 (0.08–2.56)
Current heavy daily	19.8	72.3	Ref.	17.7	Ref.	6.2	Ref.

Models adjusted for sex, age, race/ethnicity, income and educational attainment. Estimates sharing the same letter (superscripted) are statistically non-distinguishable after adjusting for sex, age, race/ethnicity, income, and education (p>0.05). AOR: adjusted odds ratio.

Table 3 displays the cessation related behaviors by group. Compared to heavy daily smoking, all other groups showed higher odds of attempting to quit smoking in the past 12 months and considering quitting smoking in the next 6 months (p<0.05), including those who started within the past 12 months (e.g. non-smoking to light daily smoking). Among smokers who made a quit attempt in the past 12 months, compared to heavy daily smokers, all other groups were generally less likely to report using nicotine replacement therapy and prescription medications in their quit attempts (p<0.05). Those who maintained light daily smoking, maintained light non-daily smoking, and those who changed from non-smoking to light non-daily smoking were also less likely to use behavioral programs in their quit attempts, while those who reduced from heavy daily to light daily smoking were more likely to use behavioral programs.

**Table 3:** Smoking cessation intention, attempts, and use of cessation aids in the past quit attempts by smoking group

*Smoking trajectory*	*Seriously considering quitting in next 6 months (N=25059)*		*Attempted to quit in past 12 months (N=25889)*		*Used nicotine replacement therapy (N=11146)*		*Used prescription medications (N=11131)*		*Used help line, counseling, clinic/class/support group, help from friends/ family, online program, or media (N=11124)*	
	*Weighted %*	*AOR (95% CI)*	*Weighted %*	*AOR (95% CI)*	*Weighted %*	*AOR (95% CI)*	*Weighted %*	*AOR (95% CI)*	*Weighted %*	*AOR (95% CI)*
Light daily to light daily	37.4^g^	1.21 (1.12–1.30)	41.2^f^	1.32 (1.22–1.42)	21.9^c^	0.73 (0.64–0.85)	10.5^d^	0.53 (0.45–0.64)	32.5^bd^	0.80 (0.71–0.90)
Heavy daily to light daily	55.2^e^	2.54 (2.23–2.90)	57.7^d^	2.58 (2.25–2.96)	26.7^ab^	0.93 (0.77–1.14)	16.1^bc^	0.84 (0.66–1.07)	44.0^a^	1.28 (1.06–1.55)
Light non-daily to light daily	53.6^e^	2.39 (1.92–2.96)	62.7^cd^	3.14 (2.53–3.90)	15.3^de^	0.49 (0.35–0.70)	9.1^de^	0.49 (0.31–0.77)	33.4^cd^	0.86 (0.67–1.09)
Heavy non-daily to light daily	46.8^efg^	1.88 (1.07–3.29)	62.7^bde^	3.31 (1.71–6.40)	19.8^bce^	0.68 (0.26–1.79)	9.2^cde^	0.53 (0.13–2.09)	44.0^acd^	1.37 (0.57–3.26)
Nonsmoker to light daily	59.5^bde^	3.03 (2.29–4.01)	68.3^bc^	4.07 (3.07–5.40)	18.5^ce^	0.60 (0.42–0.87)	12.7^abd^	0.67 (0.46–0.98)	34.5^cd^	0.85 (0.62–1.15)
Light daily to light non-daily	68.6^c^	4.48 (3.53–5.69)	74.4^b^	5.42 (4.17–7.04)	20.7^acd^	0.70 (0.51–0.97)	9.6^bd^	0.52 (0.31–0.87)	37.3^acd^	0.99 (0.72–1.35)
Heavy daily to light non-daily	80.9^a^	8.49 (5.84–12.35)	85.8^a^	11.50 (7.65–17.30)	25.7^bc^	0.84 (0.59–1.20)	19.1^ac^	0.98 (0.67–1.44)	41.9^ac^	1.19 (0.89–1.60)
Light non-daily to light non-daily	48.4^f^	1.87 (1.70–2.06)	48.5^e^	1.80 (1.62–1.99)	13.7^e^	0.41 (0.34–0.49)	6.0^e^	0.30 (0.23–0.39)	30.1^d^	0.75 (0.64–0.87)
Heavy non-daily to light non-daily	62.9^ce^	3.36 (1.96–5.78)	63.3^bd^	3.29 (2.09–5.16)	30.0^bc^	1.07 (0.57–2.01)	9.1^cde^	0.39 (0.09–1.64)	38.3^acd^	1.04 (0.57–1.90)
Non-smoker to light non-daily	65.2^cd^	3.73 (3.07–4.53)	58.9^d^	2.68 (2.25–3.20)	14.2^e^	0.42 (0.29–0.60)	9.0^de^	0.43 (0.31–0.60)	31.2^bd^	0.73 (0.56–0.95)
Light daily to heavy non-daily	69.9^acefg^	5.08 (1.17–22.12)	72.5^abdef^	5.52 (1.11–27.40)	23.5^bce^	0.74 (0.16–3.43)	0.0^f^	-	12.6^acd^	0.27 (0.05–1.56)
Heavy daily to heavy non-daily	75.8^ac^	6.42 (3.63–11.37)	71.8^bc^	4.97 (2.83–8.72)	27.3^bc^	0.96 (0.53–1.76)	14.9^cd^	0.79 (0.36–1.73)	44.4^abc^	1.35 (0.77–2.37)
Light non-daily to heavy non-daily	60.9^acef^	3.39 (1.40–8.18)	68.0^bde^	4.30 (1.76–10.47)	15.0^bce^	0.47 (0.16–1.37)	7.0^cde^	0.37 (0.09–1.46)	32.3^acd^	0.94 (0.30–2.93)
Heavy non-daily to heavy non-daily	54.1^def^	2.43 (1.64–3.59)	56.1^cde^	2.58 (1.71–3.89)	20.6^bce^	0.62 (0.32–1.20)	12.1^cde^	0.61 (0.26–1.47)	26.3^cd^	0.67 (0.36–1.24)
Nonsmoker to heavy non-daily	81.6^abc^	9.11 (3.25–25.53)	76.8^abd^	6.51 (2.35–18.04)	18.9^bce^	0.55 (0.18–1.68)	11.9^cde^	0.57 (0.10–3.30)	32.7^acd^	0.80 (0.26–2.48)
Current heavy daily	33.0	Ref.	33.9	Ref.	29.4	Ref.	19.9	Ref.	37.5	Ref.

Models adjusted for sex, age, race/ethnicity, income and educational attainment. Estimates sharing the same letter (superscripted) are statistically non-distinguishable after adjusting for sex, age, race/ethnicity, income, and education (p>0.05). AOR: adjusted odds ratio.

Table 4 displays receiving advice from healthcare providers by group among those who saw their healthcare providers. Compared to heavy daily smokers, those maintaining light daily smoking and those maintaining light non-daily smoking were less likely to be advised to set a quit date (p<0.05). Further, smokers in five groups were less likely than heavy daily smokers to be advised to use nicotine replacement therapy and prescribed a pill for smoking cessation: maintaining light daily smoking, maintaining light non-daily smoking, changing from light non-daily to light daily smoking, changing from non-smoking to light daily smoking, and changing from non-smoking to light non-daily smoking (p<0.05). Lastly, compared to heavy daily smokers, those who changed from heavy daily to heavy non-daily smoking or who maintained light non-daily smoking were less likely to be advised to use behavioral cessation programs (p<0.05).

**Table 4:** Healthcare providers’ advice and cessation medication prescription by smoking group

*Smoking group*	*Advised to use help line, class, program, or counseling (N=12230)*		*Advised to use nicotine replacement therapy (N=12276)*		*Prescribed a medication for cessation (N=12257)*		*Advised to set quit date (N=12248)*	
	*Weighted %*	*AOR (95% CI)*	*Weighted %*	*AOR (95% CI)*	*Weighted %*	*AOR (95% CI)*	*Weighted %*	*AOR (95% CI)*
Light daily to light daily	25.8^ae^	0.95 (0.84–1.07)	24.5^bdh^	0.88 (0.79–0.99)	18.5^ad^	0.72 (0.64–0.82)	15.7^cd^	0.81 (0.70–0.93)
Heavy daily to light daily	29.2^ac^	1.16 (0.95–1.43)	27.6^cde^	1.07 (0.87–1.31)	24.3^c^	1.02 (0.82–1.26)	20.9^ab^	1.16 (0.93–1.45)
Light non-daily to light daily	25.3^ad^	0.94 (0.68–1.29)	18.6^fh^	0.65 (0.46–0.92)	15.6^de^	0.64 (0.43–0.95)	20.2^abc^	1.15 (0.78–1.69)
Heavy non-daily to light daily	11.9^cde^	0.37 (0.11–1.19)	20.4^efh^	0.68 (0.17–2.76)	19.6^bcde^	0.89 (0.19–4.21)	31.3^ab^	2.34 (0.81–6.78)
Non-smoker to light daily	18.0^bde^	0.62 (0.37–1.04)	13.2^fi^	0.43 (0.26–0.73)	13.5^de^	0.50 (0.32–0.81)	13.8^bd^	0.70 (0.40–1.23)
Light daily to light non-daily	19.7^de^	0.68 (0.46–1.00)	21.7^efh^	0.77 (0.53–1.13)	19.1^cd^	0.80 (0.54–1.16)	14.5^bd^	0.75 (0.48–1.18)
Heavy daily to light non-daily	33.7^a^	1.40 (0.94–2.10)	32.1^cde^	1.26 (0.82–1.93)	35.8^b^	1.66 (1.12–2.46)	20.6^abd^	1.07 (0.69–1.68)
Light non-daily to light non-daily	21.8^d^	0.76 (0.62–0.93)	19.1^fgi^	0.63 (0.52–0.76)	13.6^e^	0.50 (0.40–0.62)	14.7^d^	0.75 (0.61–0.93)
Heavy non-daily to light non-daily	29.2^ad^	1.14 (0.52–2.53)	25.6^efh^	0.92 (0.41–2.08)	12.2^cde^	0.42 (0.12–1.50)	13.5^bd^	0.67 (0.27–1.61)
Non-smoker to light non-daily	22.2^cde^	0.80 (0.55–1.17)	14.7^fgi^	0.48 (0.32–0.73)	14.5^de^	0.54 (0.35–0.85)	17.4^bd^	0.91 (0.59–1.40)
Light daily to heavy non-daily	21.0^ad^	0.69 (0.09–5.54)	7.8^ehi^	0.19 (0.03–1.29)	0.0^f^	-	18.1^abd^	1.09 (0.13–9.10)
Heavy daily to heavy non-daily	11.7^de^	0.35 (0.13–0.97)	27.9^aegh^	0.96 (0.51–1.83)	22.1^bcde^	0.80 (0.36–1.77)	35.1^a^	2.35 (1.04–5.28)
Light non-daily to heavy non-daily	22.4^ad^	0.73 (0.19–2.78)	45.9^abcf^	2.04 (0.48–8.59)	18.2^bcde^	0.73 (0.17–3.12)	23.2^abd^	1.37 (0.39–4.78)
Heavy non-daily to heavy non-daily	24.7^ad^	0.89 (0.41–1.93)	22.8^efh^	0.74 (0.34–1.63)	32.1^abc^	1.37 (0.71–2.64)	18.7^abd^	0.98 (0.40–2.41)
Non-smoker to heavy non-daily	28.8^ad^	1.05 (0.19–5.68)	12.5^efh^	0.32 (0.03–4.05)	22.6^bcde^	0.81 (0.11–5.78)	26.3^abd^	1.51 (0.26–8.84)
Current heavy daily	26.6	Ref.	27.7	Ref.	26.1	Ref.	19.2	Ref.

Models adjusted for sex, age, race/ethnicity, income and educational attainment. Estimates sharing the same letter (superscripted) are statistically non-distinguishable after adjusting for sex, age, race/ethnicity, income, and education (p>0.05). AOR: adjusted odds ratio.

Tobacco use and cessation behaviors, as well as healthcare providers’ advice and cessation medication prescription varied significantly when comparing these variables across smoking groups engaged in light smoking or non-daily smoking at T1. For example, changes in cigarette per months differ significantly between most groups (p<0.05). Light non-daily to light non-daily smokers and non-smoking to light non-daily smokers were less likely to smoke their first cigarettes ≤30 minutes after waking up than other groups (p<0.05). Light daily to light daily smokers were one of the least likely groups to report seriously considering quitting smoking in the next six months and attempted to quit smoking in the past 12 months (p<0.05). Non-smoking to light daily smokers and non-smoking to light non-daily smokers were among the groups that were least likely to be advised to use nicotine replacement therapy. Heavy daily to light daily smokers, light non-daily to light daily smokers, heavy non-daily to light daily smokers, heavy daily to heavy non-daily smokers were more likely than other groups to be advised to set a quit date (p<0.05).

## DISCUSSION

Research on smokers who engaged in light and/ or non-daily smoking is limited, especially on the heterogeneity of smoking history within light daily and non-daily smokers, their use of non-cigarette tobacco products, and their cessation behaviors. This analysis advanced our understanding on light daily and non-daily smokers in the following ways.

First, among light daily and non-daily smokers, over two-thirds maintained light and/or non-daily use over a 12-month period (i.e. maintained either light daily, light non-daily, or heavy non-daily smoking). While some literature suggests that light daily and non-daily smoking may be a transient stage in the natural history of smoking^16^, overall the literature is mixed because both outcomes are possible depending on the characteristics of the smokers involved. In our study, we found light and/or non-daily smoking to be largely stable within a year.

Second, our findings on changes in cigarette consumption help shed light on the nuances of light daily and non-daily smoking behavior with respect to health risks. For example, despite maintaining the same smoking frequency and quantity, those who maintained light daily and light non-daily smoking showed little changes in cigarette consumption, while those who maintained heavy non-daily smoking showed an increase in cigarette consumption. Further, it was previously unclear if changing from heavy non-daily to light daily smoking represents an increase or a decline in total cigarette consumption, given the group represents a decrease in quantity but an increase in frequency. Our finding clarified that this group was associated with a reduction of over 200 cigarettes per month.

Third, in agreement with previous literature, this analysis found that light daily and non-daily smokers were more likely than heavy daily smokers to have made a quit attempt^9^ and to seriously consider quitting smoking. However, it is worth noting the heterogeneity by groups. Specifically, those who maintained light daily smoking were only slightly more likely than heavy daily smokers to seriously consider quitting smoking, perhaps due to their lower perceived risks of smoking-related illnesses^17^. Despite the intention to quit smoking, this analysis showed that smokers in several light daily and nondaily smoking groups were less likely than heavy daily smokers to be advised by healthcare providers to set a quit date, to use nicotine replacement therapy or behavioral programs, and/or be prescribed cessation medications. Given the elevated health risks associated with light daily and non-daily smoking^11-13^ and the effectiveness of brief smoking cessation interventions by healthcare providers^18^, it is important for healthcare providers to assess, advise, and assist light daily and non-daily smokers to quit smoking.

Literature related to smoking trajectories demonstrates how light daily and non-daily smoking can vary between being a transient and stable stage, while clarifying some of the characteristics associated with smokers and helping to predict how the smoking behavior of individuals changes over time. Rather than using cigarettes smoked per day as our study did, some smoking trajectory literature has shown that days smoked per month is the best outcome variable for distinguishing between the characteristics of different smoker groups that are represented in trajectories^19^. Individuals who eventually quit smoking are less likely to have been Black or broken the rules during high school and more likely to have been Hispanic and have mothers who completed higher education^19^. Alternatively, individuals who are early and established smokers are less likely to have educated mothers and be Black or Hispanic, while being more likely to be out of school or employed^19^. Additional smoking trajectory literature, measuring cigarettes per day, suggests that continuous heavy smokers and occasional smokers are more likely to experience unemployment compared to non-smokers^20^. Other smoking trajectory research has suggested that high amounts of trouble at school and low education achievement also lead to an increased probability of heavy and continuous smoking^21^. These studies somewhat correlate with our findings that smokers with less education and less income were more likely to be light daily and non-daily smokers. However, these studies also suggest that smoking cessation interventions and outreach need to differ based on racial/ethnic group since racial groups do not all necessarily display the same trends in smoking behavior.

### Strengths and limitations

While the strength of this analysis was a large sample size, which allowed examination of various smoking groups involving light daily and non-daily smoking, it also has limitations. The data were from the 2010–2011 TUS-CPS, which are slightly dated. Given the increasing trends in light daily and non-daily smoking in recent years, this analysis could have underestimated the prevalence of various groups. However, the 2010–2011 TUS-CPS was the only cycle that cessation-treatment use and healthcare provider advice were assessed, and therefore the only cycle that could answer our research questions. Nonetheless, this analysis is unable to show how the introduction of e-cigarettes in the tobacco marketplace may have influenced frequency and intensity of smoking as well as access to evidence-based cessation treatments. A threshold of 100 cigarettes as opposed to ‘ever use’ was used because smokers who did not meet this threshold in the survey were not asked detailed tobacco use questions. As a result, this threshold inadvertently might not have captured younger smokers who were just experimenting or initiating cigarette use. Another limitation of this study was the reliance on memory for baseline tobacco use status due to the absence of prospective data. Due to the cross-sectional nature of this analysis, it is important to refrain from making causal inferences based on our findings.

## CONCLUSIONS

This analysis showed that light daily and non-daily smoking could be a result of heterogenous smoking groups, while the majority involved maintaining light smoking in a daily or non-daily fashion. It also showed that non-cigarette tobacco product use was more prevalent in only some light daily and non-daily smoking groups compared to heavy daily smoking. Further, despite intention to quit smoking, smokers in several light daily and non-daily smoking groups were less likely than heavy daily smokers to be assisted by healthcare providers for smoking cessation. Prior research has also demonstrated that non-daily smokers are less likely than daily smokers to be advised to quit smoking or asked about their tobacco use by their doctors, despite having a greater desire to quit smoking than daily smokers^22^. Additionally, this phenomenon persists even after controlling for demographic covariates and other factors such as health status^22^. This finding mirrors our own results and highlights an area that physicians can improve in when treating their patients. Furthermore, educating healthcare providers about the harm of light daily and non-daily smoking may increase the smoking cessation effort among light daily and non-daily smokers.

## CONFLICTS OF INTEREST

The authors have completed and submitted the ICMJE Form for Disclosure of Potential Conflicts of Interest and none was reported.

## FUNDING

This study was supported by the Division of Intramural Research at the National Institute on Minority Health and Health Disparities, National Institutes of Health. The views presented in this article do not necessarily reflect the views of the U.S. Government, Department of Health and Human Services, National Institutes of Health, or National Institute on Minority Health and Health Disparities.

## PROVENANCE AND PEER REVIEW

Not commissioned; externally peer reviewed.
