# Supplementary material for: Heterogeneity in past-year smoking, current tobacco use, and smoking cessation behaviors among light and/or non-daily smokers
Source: Tob Induc Dis. 2020 Sep 4;18:74. doi: 10.18332/tid/125724 (PMC7516252; doi:10.18332/tid/125724)
Supplement: Supplementary file 1 [file TID-18-74-S1.pdf]

Supplementary Table S1: Adjusted odds ratios and 95% confidence intervals between demographic characteristics and smoking group as compared to heavy daily smokers

Demographics	Light daily to light daily	Heavy daily to light daily	Light nondaily to light daily	Heavy nondaily to light daily	Non-smoker to light daily	Light daily to light nondaily	Heavy daily to light nondaily	Light nondaily to light nondaily	Heavy nondaily to light nondaily	Non-smoker to light nondaily	Light daily to heavy nondaily	Heavy daily to heavy nondaily	Light nondaily to heavy nondaily	Heavy nondaily to heavy nondaily	Non-smoker to heavy nondaily
**Sex (ref= female)**															
Male	**0.48 (0.44, 0.52)**	**0.60 (0.53, 0.69)**	**0.51 (0.41, 0.64)**	0.83 (0.46, 1.50)	**0.46 (0.35, 0.59)**	**0.46 (0.37, 0.58)**	0.78 (0.58, 1.40)	0.82 (0.74, 0.91)	0.91 (0.58, 1.44)	**0.61 (0.52, 0.73)**	1.10 (0.35, 3.50)	1.34 (0.82, 2.18)	1.10 (0.61, 1.99)	**1.93 (1.23, 2.97)**	0.80 (0.37, 1.74)
**Age (ref= over 50 years old)**															
18-34	**2.09 (1.90, 2.30)**	**2.06 (1.74, 2.44)**	**3.49 (2.77, 4.39)**	2.00 (0.99, 4.04)	**2.59 (1.90, 3.52)**	**3.00 (2.30, 3.80)**	**1.75 (1.24, 2.46)**	**2.69 (2.40, 3.02)**	1.55 (0.86, 2.78)	**3.36 (2.76, 4.10)**	0.77 (0.18, 3.22)	1.26 (0.74, 2.12)	0.91 (0.33, 2.47)	0.92 (0.54, 1.56)	0.73 (0.26, 2.07)
35-49	**1.12 (1.03, 1.23)**	0.92 (0.78, 1.08)	**1.28 (1.01, 1.62)**	0.61 (0.29, 1.30)	**0.69 (0.48, 0.99)**	0.92 (0.69, 1.22)	1.22 (0.85, 1.73)	**1.33 (1.19, 1.49)**	1.18 (0.73, 1.91)	**1.13 (0.92, 1.38)**	0.38 (0.07, 2.10)	0.67 (0.37, 1.21)	**0.49 (0.25, 0.94)**	0.71 (0.42, 1.22)	0.49 (0.17, 1.41)
**Race/ethnicity (ref= NH-White)**															
Hispanic	**5.22 (4.48, 6.09)**	**1.91 (1.40, 2.62)**	**6.64 (4.75, 9.28)**	**18.44 (8.60, 39.55)**	**5.02 (3.22, 7.83)**	**8.34 (5.75, 12.10)**	1.10 (0.50, 2.38)	**9.66 (8.15, 11.43)**	**5.01 (2.37, 10.60)**	**8.84 (6.69, 11.69)**	**10.56 (1.87, 59.53)**	1.30 (0.43, 3.96)	**8.71 (2.63, 28.82)**	**2.59 (1.20, 5.60)**	**5.66 (1.41, 22.82)**
NH-Black	**3.89 (3.44, 4.40)**	**1.71 (1.35, 2.16)**	**4.71 (3.63, 6.12)**	**4.82 (1.92, 12.10)**	**2.51, (1.67, 3.79)**	**4.27 (3.15, 5.79)**	**2.18 (1.34, 3.55)**	**4.51 (3.85, 5.28)**	**3.65 (2.09, 6.40)**	**2.98 (2.05, 4.32)**	**5.87 (1.13, 30.53)**	0.83 (0.26, 2.61)	**3.05 (1.13, 8.20)**	1.90 (0.93, 3.87)	0.95 (0.07, 12.78)
NH-AI/AN/HI	**1.75 (1.30, 2.37)**	1.35 (0.74, 2.44)	**2.58 (1.31, 5.08)**	0.41 (0.03, 5.14)	0.94 (0.42, 2.10)	0.94 (0.42, 2.10)	1.92 (0.59, 6.23)	**2.69 (1.83, 3.94)**	0.85 (0.16, 4.48)	**3.69 (2.00, 6.81)**	#	1.99 (0.30, 13.05)	0.77 (0.12, 4.76)	1.93 (0.46, 8.17)	#
NH-Asian	**4.05 (3.10, 5.30)**	**2.75 (1.70, 4.44)**	**8.18 (4.77, 14.04)**	1.90 (0.13, 28.03)	2.08 (0.77, 5.62)	**5.35 (2.85, 10.05)**	1.03 (0.24, 4.52)	**3.34 (2.32, 4.81)**	0.42 (0.03, 5.84)	**2.22 (1.18, 4.17)**	#	#	5.77 (0.49, 68.67)	#	#
NH-Multi	**1.35 (1.02, 1.79)**	**2.00 (1.26, 3.16)**	0.84 (0.37, 1.89)	#	1.48 (0.60, 3.62)	0.88 (0.36, 2.77)	1.18 (0.32, 2.46)	1.11 (0.39, 3.21)	**5.32 (1.59, 17.78)**	2.10 (0.95, 4.64)	#	2.46 (0.41, 14.85)	#	0.33 (0.03, 4.18)	#
**Education Completed (ref= Bachelor's degree or higher)**															
<HS & HS education	**0.59 (0.53, 0.67)**	**0.79 (0.62, 0.998)**	**0.53 (0.38, 0.73)**	**0.42 (0.18, 0.97)**	**0.52 (0.36, 0.73)**	**0.33 (0.23, 0.48)**	0.64 (0.39, 1.06)	**0.29 (0.25, 0.33)**	0.60 (0.28, 1.30)	**0.21 (0.16, 0.28)**	0.43 (0.70, 2.55)	2.12 (0.69, 6.48)	1.88, (0.21, 16.91)	0.67 (0.31, 1.49)	**0.28 (0.09, 0.90)**
Some college	**0.70 (0.62, 0.80)**	0.82 (0.64, 1.04)	**0.63 (0.44, 0.92)**	0.65 (0.26, 1.66)	**0.61 (0.40, 0.92)**	**0.59 (0.42, 0.83)**	0.81 (0.48, 1.38)	**0.42 (0.35, 0.50)**	0.65 (0.29, 1.52)	**0.41 (0.31, 0.54)**	0.10 (0.01, 2.03)	2.06 (0.63, 6.74)	1.70 (0.15, 19.55)	**0.41 (0.18, 0.96)**	0.83 (0.26, 2.73)
Associate Degree	**0.81 (0.69, 0.96)**	0.79 (0.57, 1.10)	0.75 (0.48, 1.17)	**0.14 (0.03, 0.72)**	0.62 (0.36, 1.06)	**0.60 (0.38, 0.95)**	1.21 (0.70, 2.09)	**0.57 (0.48, 0.69)**	0.79 (0.26, 2.38)	**0.47 (0.33, 0.68)**	#	2.01 (0.46, 8.87)	1.44 (0.10, 21.74)	0.70 (0.28, 1.76)	0.27 (0.05, 1.62)

**Family Income (ref= $75,000 or higher income)**															
<$25K	**0.89 (0.79, 0.99)**	1.25 (0.998, 1.57)	1.12 (0.82, 1.54)	0.90 (0.37, 2.23)	0.81 (0.57, 1.15)	1.13 (0.78, 1.64)	1.30 (0.81, 2.12)	**0.69 (0.60, 0.80)**	0.86 (0.39, 1.86)	**0.69 (0.53, 0.89)**	0.64 (0.04, 11.25)	0.66 (0.33, 1.31)	1.75 (0.37, 8.33)	0.94 (0.51, 1.75)	1.70 (0.67, 4.29)
$25K-<$50K	**0.89 (0.80, 0.99)**	0.98 (0.76, 1.27)	0.74 (0.54, 1.03)	1.23 (0.49, 3.08)	0.97 (0.69, 1.37)	1.09 (0.77, 1.55)	1.09 (0.69, 1.73)	**0.68 (0.59, 0.79)**	0.84 (0.39, 1.85)	**0.63 (0.49, 0.82)**	1.99 (0.13, 31.59)	0.96 (0.47, 1.93)	0.63 (0.11, 3.64)	0.54 (0.26, 1.13)	0.85 (0.25, 2.83)
50K-<$75K	0.95 (0.83, 1.09)	0.92 (0.70, 1.21)	0.86 (0.60, 1.24)	0.72 (0.18, 2.80)	0.75 (0.49, 1.14)	0.96, (0.63, 1.47)	1.36 (0.83, 2.25)	**0.76 (0.65, 0.90)**	1.11 (0.50, 2.46)	0.84 (0.61, 1.15)	1.93 (0.11, 32.96)	0.60 (0.23, 1.56)	1.91 (0.33, 11.19)	0.54 (0.27, 1.10)	0.83 (0.16, 4.36)

Estimates were obtained using a weighted multinomial regression model adjusting for all variables listed in the table. Bolded estimates are statistically significant ($p<0.05$). #Estimate not reported due to small sample.

© 2020 Omole T. et al.
